# Is lingual fracture pattern in sagittal split osteotomy associated with recovery of neurosensory disturbance?

**DOI:** 10.4317/medoral.27136

**Published:** 2025-04-06

**Authors:** Yusuf Nuri Kaba, Cemil Eren, Ahmet Emin Demirbas, Bahadır Gökberk Yaşar, Emrah Soylu, Nizami Gayıbov

**Affiliations:** 1 CID ID: 0000-0001-9221-4599. Associate Professor, Department of Oral and Maxillofacial Surgery, Faculty of Dentistry, Bursa Uludağ University; 2 ORCID ID: 0000-0003-3787-4564. Research Assistant, Department of Oral and Maxillofacial Surgery, Faculty of Dentistry, Erciyes University; 3 ORCID ID: 0000-0002-2602-6415. Associate Professor, Department Head, Department of Oral and Maxillofacial Surgery, Faculty of Dentistry, Erciyes University; 4 ORCID ID: 0000-0001-5307-5020. Research Assistant, Department of Oral and Maxillofacial Surgery, Faculty of Dentistry, Erciyes University; 5 ORCID ID: 0000-0002-9828-5096. Associate Professor, Department of Oral and Maxillofacial Surgery, Faculty of Dentistry, Erciyes University. Dentbiochem Co., Erciyes Teknopark, Kayseri, Türkiye; 6 ORCID ID: 0009-0008-3647-8176. Research Assistant, Institute of Health Science, Department of Oral and Maxillofacial Surgery, Faculty of Dentistry, Erciyes University

## Abstract

**Background:**

Bilateral sagittal split osteotomy (BSSO) is commonly used to correct mandibular deformities but may cause neurosensory disturbance (NSD) of the lower lip due to potential injury to the inferior alveolar nerve (IAN). The purpose of this study was to evaluate the effect of LSS 3 on postoperative neurosensory disturbances. The hypothesis of this study was that the LSS 3 split pattern would extend the recovery time of neurosensory disturbance.

**Material and Methods:**

The retrospective cohort study included patients who underwent BSSO surgery in Erciyes University, Oral and Maxillofacial Surgery Hospital. The primary predictor variable was the lingual split pattern. The split patterns were categorized using the Lingual Split Scale (LSS). The primary outcome was NSD. The postoperative sensation was assessed using a visual analogue scale (VAS). The secondary outcome was intraoperative nerve exposure. The nerve exposure was classified as No nerve encountered, Embedded in distal segment, Embedded in proximal segment and Nerve transected. All data were analyzed using Turcosa Cloud statistical software (Turcosa Ltd. Co., Turkey). *p*<0.05 was considered significant.

**Results:**

The study included 101 patients with 202 split sides. LSS 1 was the most common pattern (63.37%), followed by LSS 3 (25.74%). In LSS 3 split pattern, the inferior alveolar nerve mostly embedded in the proximal segment (*p*<0.05). NSD was highest in LSS 3 cases, particularly in the first 6 months postoperatively (*p*<0.05). However, no significant differences were observed after 12 months.

**Conclusions:**

LSS 3 splits may significantly increase embedded in the proximal segment and can associated with higher postoperative NSD, particularly in the first 6 months. Surgeons should consider factors contributing to LSS 3 patterns to reduce the risk of NSD.

** Key words:**Sagittal split osteotomy, lingual split patterns, neurosensory disturbance.

## Introduction

Bilateral sagittal split ramus osteotomy (BSSO) is a successful surgical procedure commonly used to correct mandibular dentoskeletal deformities (1). BSSO has many advantages such as providing better masticatory function, improving facial aesthetics, reducing temporomandibular joint pain and treating obstructive sleep apnea (2). The procedure was first introduced by Trauner and Obwegeser in 1957 and since then several modifications have been developed by Dal Pont, Hunsuck, Epker and Wolford to reduce postoperative complications and improve the stability of the treatment outcome (3). However, minimising the risk of unfavourable fractures of mandibular fragments and injury to the inferior alveolar nerve (IAN) is still technically challenging.

Neurosensory disturbance (NSD) of the lower lip due to IAN injuries causes significant morbidity and discomfort in patients (4). The incidence of this has been reported to be 9-84.6% (5). There are studies in the literature to investigate the causes of neurosensory disturbances after BSSO. Multiple factors are thought to be responsible for the development of NSD after BSSO, including fixation methods, patient age, surgical procedures, inaccurate splinting, amount of mandibular movement, surgeon experience, and timing of postoperative neurosensory evaluation (6). Even with successful and careful surgery, injury of the nerve can be unpredicTable. With all these, it is thought that the split patterns of the mandible may influence the occurrence of postoperative NSD. Reduction of unfavorable fracture and inferior alveolar nerve injury requires control of the split stage of the mandible during bilateral sagittal split osteotomy (7).

The fracture patterns of some areas, such as the lingual side of the mandibular ramus, cannot be clearly controlled during surgery. Cone beam computed tomography (CBCT) provides clear visualization of lingual fracture lines on the buccal and lingual surfaces of the mandible. In an observational study, Plooij *et al*. evaluated whether CBCT is a useful tool to analyze the fracture line in BSSO (8). In the cited study, lingual split patterns of BSSO were categorized first time. They reported that lingual split scale 3 (LSS 3, lingual split pattern along the mandibular canal) might be more at risk for nerve damage (8). Hu *et al*. evaluated lingual split patterns after BSSO using 3D reconstruction of CBCT and found no relationship between lingual split patterns and postoperative NSD (9). However, the relationship between lingual split types and NSD was not evaluated in this study. Similarly, G. Martinez-de la Cruz *et al*. reported that there was no relationship between lingual split pattern and long-term NSD (2). In this study, BSSO was applied according to the Dalpont technique and has a relatively small sample size.

In this study, the authors present the relationship between lingual split pattern and NSD after BSSO. The hypothesis of this study was that the LSS 3 split pattern would extend the recovery time of Neurosensory Disorders. The purpose of this study was to evaluate the effect of LSS 3 on postoperative neurosensory disturbances.

## Material and Methods

- Study Design and Sample

The retrospective cohort study included patients who underwent BSSO operation in Erciyes University Faculty of Dentistry, Department of Oral and Maxillofacial Surgery. This study was approved by the Local Ethics Committee for Clinical Research of Erciyes University (approval number: 2023/511). The inclusion criteria were patients who underwent BSSO due to dentofacial deformity, patients with preoperative and postoperative CBCT images, and postoperative CBCT images taken within 1 month postoperatively. The exclusion criteria were as follows patients with missing postoperative CBCT images or examination records, simultaneous genioplasty, postoperative CBCT images obtained after the 1st postoperative month, a history of facial trauma, orthognathic surgery, and maxillofacial pathology. All participants were informed about the medications, surgical procedures, and possible complications and written informed consent was obtained.

- Study Variable

**Figure 1:**
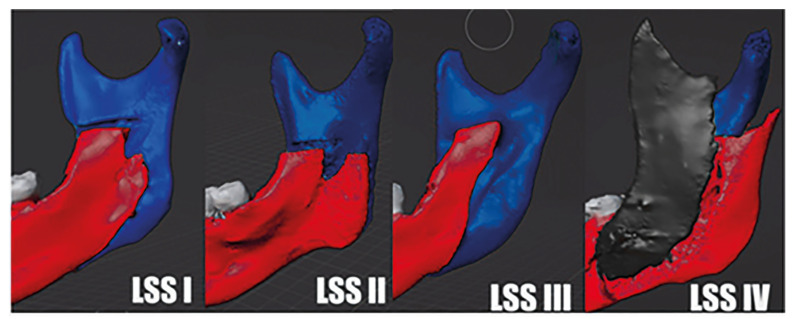
3D reconstruction of types of lingual split pattern. LSS type 1: vertical pattern of fracture line to the inferior border of the mandible (‘true’ Hunsuck). LSS type 2: horizontal pattern of fracture line to the posterior border of the ramus. LSS type 3: fracture line through the mandibular canal to the inferior border of the mandible. LSS type 4: other patterns (i.e. buccal plate fracture or bad split).

Predictor variables: The primary predictor variable was the lingual split pattern to categorize the different split patterns, a lingual split scale (LSS) was used which was developed by Plooji *et al*. (8). LSS is divided into 4 categories according to the fracture line on the lingual side of the ramus (Fig. 1). LSS 1 is a fracture line perpendicular to the inferior border of the mandible as defined by Hunsuck (10), LSS 2 is a horizontal pattern of fracture line to the posterior border of the ramus, LSS 3 is a fracture line through the mandibular canal to the inferior border of the mandible, and LSS 4 includes other fracture lines, i.e. bad split (8).

Primary outcome: The primary outcome was NSD. The postoperative sensation was assessed using a visual analog scale (VAS). Paresthesia was scored by asking the patient, with a minimum score of 0 and a maximum score of 100. ‘0’ means full sensation, ‘100’ means full numbness.

**Figure 2:**
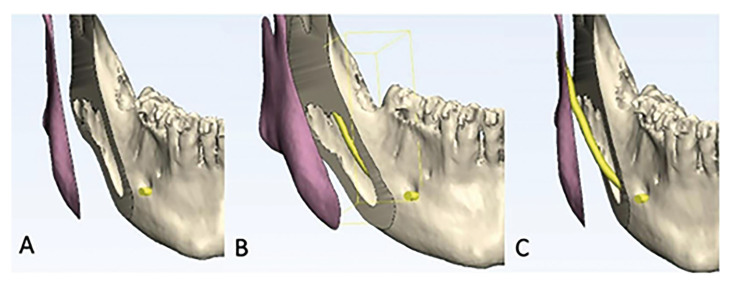
Intraoperative nerve exposure is shown in the image. A; No nerve encountered, B; Embedded in distal segment, C; Embedded in proximal segment.

Seconder outcome: The secondary outcome was intraoperative nerve exposure, which was classified using a modified classification by Lee *et al*. (11). The exposure of the inferior alveolar nerve was assessed immediately after mandibular splitting. The nerve exposure was classified as No nerve encountered; the nerve was completely embedded and not exposed, Embedded in distal segment; the nerve was visible but present in the distal segment, Embedded in proximal segment; the nerve was visible but present in the proximal segment (Fig. 2), and Nerve transected.

Covariates: The covariates were age, sex, side of the mandible, skeletal malocclusion, and amount of mandibular movement. The amount of mandibular movement was classified as less than 7 mm and more than 7 mm according to Demirbas *et al*. (3).

- Surgical Procedure

All patients were taken under general anesthesia by the same anesthesia team and operated by the same surgical team led by A.E.D at Erciyes University, Department of Oral and Maxillofacial Surgery. BSSO was performed according to the Hunsuck modification. All osteotomized fragments were fixed with mini-plates and monocortical screws. Steroids were used on a routine basis in order to resolve postoperative edema. To prevent postoperative infection and pain, all patients received antibiotics and analgesics after surgery.

- Data Analysis

The normal distribution of the data was assessed using histograms, Q-Q plots, and the Shapiro-Wilk test. Descriptive statistics were calculated for each variable. The homogeneity of variance was assessed using Levene's test. Student T-test and Mann-Whitney U-test are used to compare quantitative data between 2 groups. One-way analysis of variance and Kruskal-Wallis tests were utilized for quantitative variables in more than two groups. Pearson chi-square test was used to compare categorical data. All data were analyzed using Turcosa Cloud statistical software (Turcosa Ltd. Co., Turkey). *p*<0.05 was considered significant.

## Results

The records of 145 patients who underwent bilateral sagittal split osteotomy were scanned. 44 patients were excluded due to missing records. The study was completed by evaluating 202 split sides in 101 patients. Descriptive data regarding demographic variables are given in Table 1. The mean age was 22.60±5.34 years. Sixty-one (60.4%) of the patients were female and 40 (39.6%) were male. 28(27.72%) patients had skeletal class 2 anomaly and 73(72.28%) patients had skeletal class 3 anomaly.

The descriptive data of the split side-related variable is given in Table 2. Mean mandibular activation was 4.33±2.57 mm for 202 split sides. It was > 7 mm on 18 (8.91%) split sides and ≤ 7 mm on 184 (91.09%) split sides. The most common lingual split pattern was LSS 1, observed in 128 sides (63.37%). This was followed by LSS 3 in 52 sides (25.74%), LSS 2 in 11 sides (5.45%), and LSS 4 in 11 sides (5.45%). The mean VAS score (69.65±39.17) was highest in the postoperative 1st week. The lowest mean VAS score (8.56±20.71) was observed in the 12th month. There was no statistically significant relationship between mean mandibular activation amount and mean VAS score at any time point.

The distribution between LSS and VAS scores is given in Table 3. LSS 3 showed the highest VAS scores at all time points, with a statistically significant difference compared to LSS Type 1 at the 1st, 3rd, and 6th months (*p*<0.05). No significant difference in VAS scores was found between LSS 1 and LSS 2 at any time point (*p*>0.05). LSS 4 showed the lowest mean VAS score at all time points. In the 12th month, no statistically significant difference was observed between LSS and VAS scores (*p*>0.05).

The relationship between LSS and the intraoperative nerve exposure is shown in Table 4. The most common occurrence of embedded in the distal segment was in LSS1 with 53 (41.41%) sides, followed by 37 (28.91%) sides where the nerve was not encountered. In LSS3, the nerve was embedded in the proximal segment on 27 (51.92%) sides, followed by embedment in the distal segment. All three transected nerves were found on the sides showing LSS3. The LSS2 nerve was primarily embedded in the proximal segment, while the LSS4 nerve was not encountered. The statistical analysis revealed a significant relationship between the LSS and the intraoperative nerve exposure (*p*=0.009).

## Discussion

BSSO is a commonly used orthognathic surgical procedure for correcting mandibular deformities (12). Despite its long-standing utility and safety, complications such as unfavorable fractures and injury to the inferior alveolar nerve still occur (13). Optimal control of mandibular fracture and split during BSSO is necessary to reduce unfavorable fracture and nerve damage (7). However, direct visual control of the fracture patterns at the lingual side of the ramus is impossible during surgery (9). Plooij *et al*. classified lingual splits involving the mandibular canal as LSS3. They reported that LSS 3 may be more at risk for nerve damage (8). Since the LSS3 split pattern involves the mandibular canal, it may increase the likelihood of the nerve being embedded in the proximal segment. At the same time, postoperative neurosensory disturbance may result in extended recovery time. According to the results of the study, there was a statistical correlation between patients with LSS3 split pattern and the likelihood of the nerve being embedded in the proximal segment. At the same time, the presence of NSD in patients with LSS3 split pattern was more evident in the first 6 months.

Plooij *et al*. developed a lingual split scale to categorize different segmentation models into 4 categories (LSS 1, true Hunsuck; LSS 2, fracture line to the posterior border of the ramus; LSS 3, through to mandibular canal; LSS 4, unfavorable fracture pattern). In their study, they showed that approximately 50% of the 80 BSSO sites have LSS 1, followed by LSS 3 (8). Kaba *et al*. evaluated the lingual split pattern of 312 sites and reported that 65% were LSS 1 and 26% were LSS 3 (14). In our study, similar to the literature, approximately 63% of the 202 BSSO sides were LSS 1 followed by LSS 3 with approximately 26%. During BSSO, it is crucial to examine the lingual split to avoid unfavorable fractures or nerve damage (15). Especially in LSS 3 splits, intraoperative nerve exposure may increase. Martinez *et al*. defined a new lingual split scale and analyzed its effects on NSD and reported that postoperative NSD lasted longer in the type of split called 'Long Split' corresponding to LSS2 (2). In this study, patients were operated according to the Dalpont technique and therefore split technique compatible with LSS 2 was more common. Hu *et al*. analyzed 546 split sides and created a new split pattern classification. Accordingly, they showed that type 1 (Hunsuck type split) occurred most frequently, followed by type 4 and type 3 (split type involving the mandibular canal and foramen). They reported that the lingual split pattern was not statistically significantly associated with NSD (9). However, the relationship between lingual split types and NSD was not evaluated in this study. Present study, LSS 3 split was seen in approximately 26% of osteotomy sites and the nerve was embedded in the proximal segment in approximately 52% of patients with LSS 3 split.

Studies evaluating the relationship between lingual split patterns and NSD do not assess intraoperative exposure of the nerve. Also, the relationship between lingual split types involving the canal and NSD has not been demonstrated. Some studies in the literature have evaluated the effect of nerve manipulation on NSD and have observed a relationship between nerve manipulation and an increased risk of NSD, particularly in cases dissected from the proximal segment (16). In addition, manipulation seems to be an important risk factor for nerve laceration (17). In the study by Kuhlefet *et al*. evaluating the degree of nerve manipulation, 50 of a total of 82 nerves were exposed, 8 had to be dissected from the underlying bone and 4 were lacerated during the procedure. They reported that increasing the degree of manipulation increased the risk of NSD (18). Demirbas *et al*. evaluated the relationship between nerve manipulation and NSD and reported that the risk of NSD and recovery time increased with increasing degree of manipulation (3). In this study, among 52 sides displaying LSS type 3 split, the nerve was embedded in the proximal segment in 27 (51.92%) sides, and nerve transection was observed in 3 (5.77%) sides. Additionally, VAS scores were statistically significantly higher on LSS type 3 sides at the 1st week, 1st month, 3rd month, and 6th month (*p*<0.05). However, at the 12th month, although the VAS score remained higher on sides with LSS type 3 split, the difference was not statistically significant (*p*>0.05). The results of this study indicate that the nerve is more frequently embedded in the proximal segment on sides with LSS type 3, leading to a significant increase in postoperative VAS scores (*p*<0.05). Plooij *et al*. reported that medial horizontal osteotomy terminating in front of the lingula increased the possibility of LSS 3 (8). Kaba *et al*. found that osteotomy ending in front of the lingula had no effect on the splitting pattern (14). Posnick *et al*. described low and short osteotomy which reduced unfavorable splitting without decreasing functional sensory recovery of the IAN. Low and short osteotomy may increase the likelihood of a split pattern involving the mandibular canal and the nerve remaining in the proximal segment. Susarla *et al*. also reported that low osteotomy and the nerve remaining in the proximal segment did not make a difference in terms of NSD at the end of the 1st year (19). However, these studies did not include a control group. In this study, there was no statistically significant difference in terms of NSD in LSS groups like Susarla at the end of the 1st year. However, in LSS type 3, VAS scores were higher at all time points when nerve transection occurred or when the nerve was embedded in the proximal segment. This result shows that the split pattern temporarily increases the NSD in the first period of healing.

Patients' age, sex, or the amount of movement of their jaws, as well as nerve damage, can also influence the risk of NSD after surgery. Nishioka *et al*. showed a correlation between the increase in the incidence of NSD and patient age (20). On the contrary, there are studies suggesting that there is no relationship between increasing age and the occurrence of NSD after BSSO. Bruckmoser *et al*. reported that there was a higher incidence of NSD formation after BSSO in women (1). However, most studies have reported that sex does not affect the occurrence of NSD (21). In our study, no statistically significant relationship was found between sex, age, and NSD (*p*<0.05). This may be since the majority of our patients were between 18-30 years of age. In cases of mandibular advancement, nerve tension may cause damage to the IAN, but it is unlikely to result in permanent damage. Studies have reported that the risk of NSD after setback surgery is lower than after advancement surgery. Furthermore, a substantial correlation has been found between dentoskeletal deformities of class 2 and class 3. In this study, no correlation was found between skeletal deformity and NSD (*p*>0.05). Excessive mandibular movement is reported to be an important risk factor contributing to the development of NSD in BSSO (22). Movements of 7mm or more increase IAN tension and compression, possibly increasing NSD (3,22). Kuhlefet *et al*. reported that there was no relationship between the amount of mandibular movement and NSD in their study (18). In our study, no statistically significant difference was found between the amount of mandibular movement and postoperative VAS (*p*>0.05).

This study has some limitations. Firstly, the evaluation of NSD along the inferior alveolar nerve distribution can be carried out using subjective, relatively objective, and purely objective methods. Chen *et al*. reported a good correlation between subjective assessment and 2-point discrimination (an objective test) (23,24). There are also studies in the literature that show a positive correlation between subjective assessment methods when compared with objective assessment methods (25,26). We thought that the patient could determine whether there was a change in sensitivity. Therefore, in this study, we chose to use subjective assessment to evaluate NSD after sagittal split osteotomy. Second, there is a risk of IAN nerve injury at various stages of BSSO. Nerve injury may occur during soft tissue dissection medial to the mandibular ramus, dissection of the nerve from the proximal segment, osteotomy, and fixation of osteotomy fragments (3,16,27). Due to the retrospective nature of the study, we could not evaluate all of these factors.

In conclusion, a relationship was found between LSS 3 split pattern and embedment in the proximal segment. At the same time, LSS 3 significantly increases the mean VAS value due to NSD in the first 6 months. LSS 3 split pattern may extend postoperative NSD recovery time compared to other split patterns. After 12 months, no significant difference was found between LSS type and mean VAS. It is recommended that surgeons performing orthognathic surgery should consider the factors that increase the LSS 3 split pattern.

## Figures and Tables

**Table 1:** Descriptive data of the demographic variable.

Variable		Total n=101
Age(years)		22.60±5.34
Sex	Female	61(60.4)
	Male	40(39.6)
Deformity	Class 2	28(27.72)
	Class 3	73(72.28)

Data was expressed as n(%), mean ±standard deviation.

**Table 2:** Descriptive data of each variable for two split sides.

Variable		n=202
Total Mandibular Activation (mm)		4.33±2.57
Type of Activation	>7mm	18(8.91)
	≤7mm	184(91.09)
Lingual Split Pattern	LSS 1	128(63.37)
	LSS 2	11(5.45)
	LSS 3	52(25.74)
	LSS 4	11(5.45)
Degree of Nerve Injury	VAS 1^st^ week	69.65±39.17
	VAS 1^st^ month	33.54±35.37
	VAS 3^rd^ month	19.55±28.34
	VAS 6^th^ month	11.49±21.96
	VAS 12^th^ month	8.56±20.71

Data was expressed as n(%), mean ±standard deviation.

**Table 3:** Distribution of VAS value in the LSS group.

VAS	Lingual Split Pattern				*P*
	LSS 1 (n=128)	LSS 2 (n=11)	LSS 3 (n=52)	LSS 4 (n=11)	
1^st^ week	67.73±39.84 90(32.5-100)^ a^	70.90±42.29 100(40-100)^ a^	81.53±31.70 100(70-100)^ a^	34.54±40.53 20(0-80)^ b^	0.003 ^ƞ^
1^st^ month	29.29±33.16 20(0-57.5)^ a^	20.99±22.11 20(0-30)^ ab^	50.09±37.99 50(16.25-87.5)^ b^	17.27±34.66 0(0-20)^ a^	0.001^ƞ^
3^rd^ month	16.68±25.07 0(0-30)^ a^	14.55±19.16 10(0-20)^ ab^	30.10±35.32 12.5(0-57.5)^ b^	8.18±24.01 0(0-0)^ a^	0.017^ƞ^
6^th ^month	9.02±17.88 0(0-10)^ a^	10.91±12.21 10(0-20)^ a^	19.13±30.74 0(0-27.5)^ b^	4.54±15.06 0(0-0)^ a^	0.045^ƞ^
12^th^ month	6.53±16.40 0(0-0)	5.45±8.20 0(0-10)	15.48±30.38 0(0-18.75)	2.73±9.04 0(0-0)	0.132^ƞ^

Data are expressed as median (first-third quartile) and mean ±standard deviation. ^ƞ^,Kruskal-Wallis test<a.

**Table 4:** Relationship between lingual split pattern and intraoperative nerve exposure.

Variable		Nerve not encountered (n=52)	Embedded in the distal segment (n=73)	Embedded in the proximal segment (n=74)	Nerve transected (n=3)	P
Lingual Split Pattern	LSS1	37(28.91)	53(41.41)	38(29.68)	0(0)	0.009 ^ƞ^
	LSS2	3(27.27)	3(27.27)	5(45.45)	0(0)	
	LSS3	7(13.46)	15(28.85)	27(51.92)	3(5.77)	
	LSS4	5(45.45)	2(18.18)	4(36.36)	0(0)	

Data was given n(%). ^ƞ^: Kruskal-Wallis test.
